# Identification of Tumor Mutation Burden and Immune Infiltrates in Hepatocellular Carcinoma Based on Multi-Omics Analysis

**DOI:** 10.3389/fmolb.2020.599142

**Published:** 2021-02-16

**Authors:** Lu Yin, Liuzhi Zhou, Rujun Xu

**Affiliations:** ^1^ Department of Pathology, Affiliated Hangzhou First People’s Hospital, Zhejiang University School of Medicine, Hangzhou, China; ^2^ Department of Surgery, The Second Affiliated Hospital, Zhejiang University School of Medicine, Hangzhou, China

**Keywords:** hepatocellular carcinoma, immune infiltration, tumor mutation burden, biomarkers, prognosis

## Abstract

We aimed to explore the tumor mutational burden (TMB) and immune infiltration in HCC and investigate new biomarkers for immunotherapy. Transcriptome and gene mutation data were downloaded from the GDC portal, including 374 HCC samples and 50 matched normal samples. Furthermore, we divided the samples into high and low TMB groups, and analyzed the differential genes between them with GO, KEGG, and GSEA. Cibersort was used to assess the immune cell infiltration in the samples. Finally, univariate and multivariate Cox regression analyses were performed to identify differential genes related to TMB and immune infiltration, and a risk prediction model was constructed. We found 10 frequently mutated genes, including TP53, TTN, CTNNB1, MUC16, ALB, PCLO, MUC, APOB, RYR2, and ABCA. Pathway analysis indicated that these TMB-related differential genes were mainly enriched in PI3K-AKT. Cibersort analysis showed that memory B cells (*p* = 0.02), CD8+ T cells (*p* = 0.09), CD4+ memory activated T cells (*p* = 0.07), and neutrophils (*p* = 0.06) demonstrated a difference in immune infiltration between high and low TMB groups. On multivariate analysis, GABRA3 (*p* = 0.05), CECR7 (*p* < 0.001), TRIM16 (*p* = 0.003), and IL7R (*p* = 0.04) were associated with TMB and immune infiltration. The risk prediction model had an area under the curve (AUC) of 0.69, suggesting that patients with low risk had better survival outcomes. Our study demonstrated for the first time that CECR7, GABRA3, IL7R, and TRIM16L were associated with TMB and promoted antitumor immunity in HCC.

## Introduction

Hepatocellular carcinoma (HCC) is the seventh most common cancer worldwide and the fourth leading cause of cancer-related deaths (Yang et al., 2019). Despite the efforts made by the developing countries in the past few years, 80% of HCC cases are from these countries (Yang et al., 2019). HCC is the main type of primary liver cancer, accounting for more than 80% of the cases (El-Serag and Rudolph, 2007). In America, the incidence of HCC increased in each consecutive birth cohort through 1959, predominantly in Asian Americans. The incidence of HCC dropped significantly from 1960 to 1969 birth cohorts, and it was expected that the incidence would drop further from 2013 to 2020, with about 1.59% decline in men and 2.20% decline in women. However, the epidemiological data based on the population cancer register show that the incidence of HCC has increased 4-fold in the past 40 years, and this increase in the incidence will continue in the future (Petrick et al., 2016; Massarweh and El-Serag, 2017; Singal et al., 2020). The common treatment methods for HCC include surgery, radiotherapy, chemotherapy, and immunotherapy. Despite the various treatment methods, the overall survival rate of HCC is low and the mortality rate is high. The 10-year survival rate is approximately 10%, and it is accompanied by high recurrence and metastasis (Siegel et al., 2016).

In recent years, immunotherapy has become a common treatment for metastatic and aggressive tumors (Thyer et al., 2013; Tian et al., 2019). Tumor immunotherapy principally refers to a treatment that uses the body’s own immune system to attack the cancer cells. Immunotherapy mainly covers tumor vaccines, biological therapy, CAR-T cells, and immune checkpoint inhibitors (PD-1, CTLA-4) (Prieto et al., 2015). Kim et al. reported that inhibiting PD-1/PD-L1 increased the infiltration of CD8+ T cells resulting in the killing of the tumor cells (Kim et al., 2018). PD-1 checkpoint inhibitors such as *atezolizumab* and *nivolumab* have been shown to significantly improve the clinical symptoms of patients and prolong survival time in non-small cell lung cancer (Chardin et al., 2020), renal cell carcinoma (Buchbinder et al., 2019), breast cancer (Szekely et al., 2018), and melanoma (Luke et al., 2017). However, Chiu’s study demonstrated that upregulation of PVRL1 inhibited the cytotoxic T cell response through the T-cell immunoglobulin and ITIM domain (TIGIT), thus mediating resistance to PD1 inhibitors in HCC (Chiu et al., 2020). Immunotherapy is only effective in about one-fifth of the patients, and most patients are unable to benefit from it.

Tumor mutation burden (TMB) is a recent biomarker that is used to predict the effect of immunotherapy. This refers to the total number of mutations per megabase in the genome. Generally speaking, the higher the TMB, the greater the difference in the tumor tissue, and the higher the patients benefit from immunotherapy (Chan et al., 2019). Previous studies have indicated that a high TMB predicts a better prognosis in melanoma and non-small cell lung cancer (Chen et al., 2019a; Chen et al., 2019b). However, research on TMB and immune infiltration in hepatocellular carcinoma remains inconclusive.

In this study, we collected somatic mutation data and transcriptome data from The Cancer Genome Atlas (TCGA) database. We aimed to explore the TMB combined with immune infiltrates in HCC. Our study demonstrated that *CECR7*, *GABRA3*, *IL7R*, and *TRIM16L* were associated with TMB and promoted antitumor immunity in HCC.

## Materials and Methods

### Data Acquisition and Processing

Transcriptome data, somatic mutation data, and clinical information of 374 HCC samples and 50 matched normal or adjacent tissue samples were obtained from the GDC portal (https://portal.gdc.cancer.gov/). In addition, the clinical data of HCC patients (n = 376) comprising age, sex, survival time, survival status, grade, stage, and the American Joint Committee on Cancer (AJCC-TNM) staging were collected (Table 1).

**TABLE 1:** Basic clinical information of all 376 HCC patients from TCGA cohort.

Variables	TCGA cohorts
	(n = 376)
Age	59.45 ± 13.49
Gender	
Female	122 (32.4%)
Male	254 (67.6%)
Tumor Grade	
G1/G2	235 (62.5%)
G3	123 (32.7%)
G4	13 (3.5%)
Unknow	5 (1.3%)
Pathologic Stage	
I&II	261 (69.4%)
III&IV	91 (24.2%)
Unknow	24 (6.4%)
AJCC-T	
T1	185 (49.2%)
T2	94 (25.0%)
T3	81 (21.5%)
T4	13 (3.5%)
Unknow	3 (0.8%)
AJCC-N	
N0	257 (68.4%)
N1-N3	4 (1.1%)
Unknow	115 (30.6%)
AJCC-M	
M0	272 (72.3%)
M1	4 (1.1%)
Unknow	100 (26.6%)

### Classification of Clinical Data Based on the Tumor Mutation Burden

We calculated TMB by the total number of mutations per megabase of the genome. We processed the TMB and raw transcriptome data obtained from the GDC portal using the R software (https://www.r-project.org/) and Excel “vlookup” to obtain the TMB scores of each sample. According to the median of the TMB scores (median = 2.45), we divided the HCC samples into high and low TMB groups. In addition, We analyzed the relationship between TMB scores and clinical characteristics (Supplementary Figure S1).

### Multi-Omics Analysis

The somatic mutation data processed by Varscan were downloaded from TCGA, and the “maftools” package (http://bioconductor.org/) was used to visualize the TMB information (Koboldt et al., 2012). Based on the Java working environment, strawberry-perl (https://www.perl.org/) was used to run the perl script to extract the genome mutation information of the 364 HCC patients.

Using the R language, we merged the mutation information of HCC patients with the clinical survival data. According to the median value of the TMB scores, samples were classified into high and low TMB groups. Gene ontology (GO) and gene set enrichment analysis (GSEA) were performed as previously described (Subramanian et al., 2005). A p-value < 0.05 and |log(fold change)>1| were regarded to denote enrichment significance. Cibersort is a deconvolution algorithm used to evaluate the relative abundance of immune infiltration in clinical samples. It is an analytical tool from the Alizadeh Lab developed by Newman et al. to provide an estimation of the abundances of member cell types in a mixed cell population, using gene expression data (Newman et al., 2015). A threshold value of *p* < 0.05 and 1,000 repeated calculations were considered as successful.

Uni and multivariate Cox regression analyses were conducted to assess the immune-related genes. Based on the results of the multivariate regression analysis, we constructed a risk prediction model and divided the patients into high-risk and low-risk groups according to the risk scores (TMBRS). A receiver operating characteristic (ROC) curve was used to test the sensitivity and specificity of this model. Finally, an analysis of immune cell infiltration with survival outcomes was conducted using the Kaplan–Meier method.

### Statistical Analysis

All statistical analyses were performed using R, including the Kaplan-Meier survival analysis and uni- and multivariate Cox regression analyses. For all comparisons, *p* < 0.05 was considered to indicate statistical significance.

## Results

### Landscape of Frequent Gene Mutations in Hepatocellular Carcinoma

**FIGURE 1:**
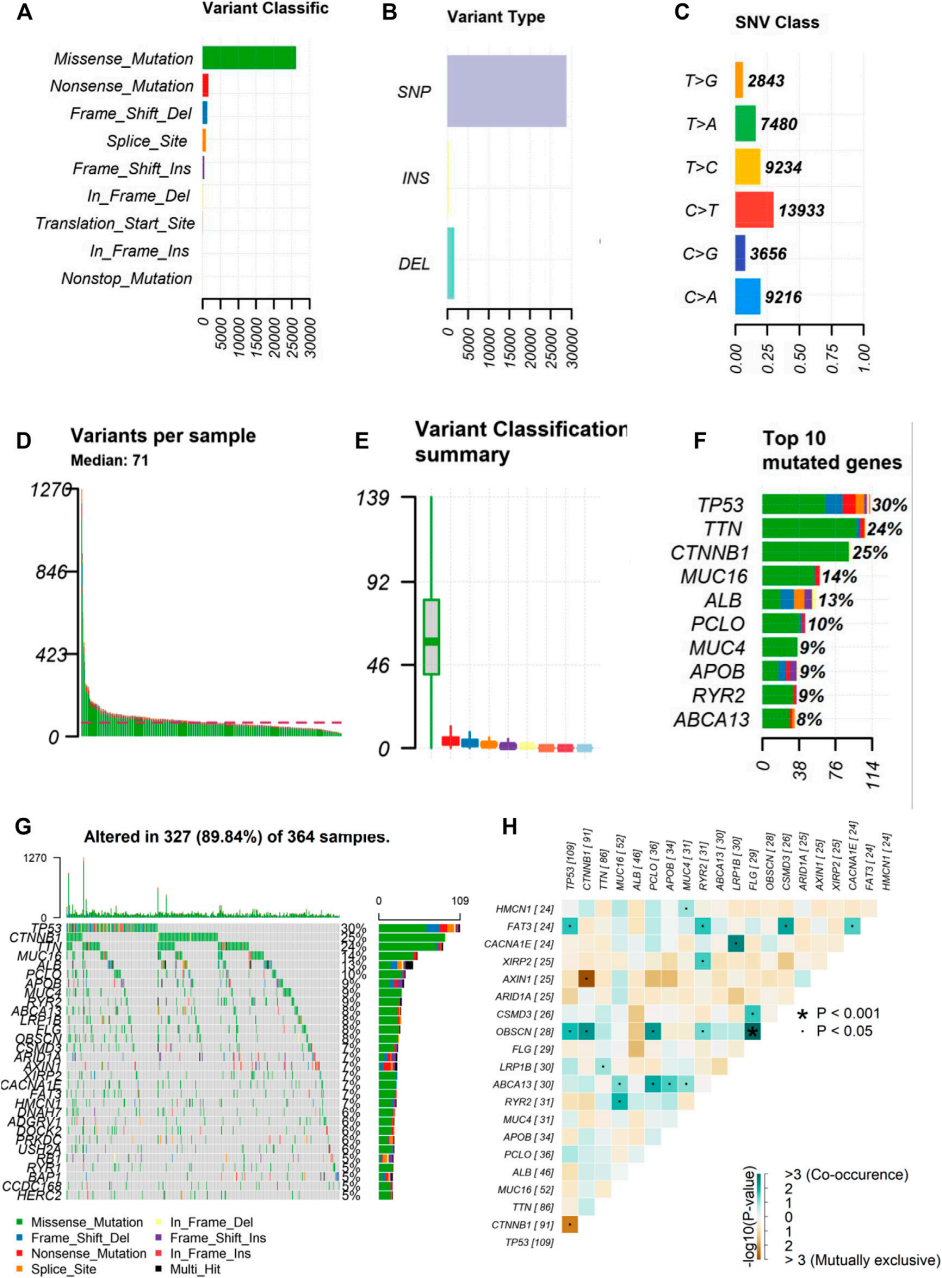
Landscape of frequently gene mutation in HCC. **(A–C)** Statistical calculations of mutation types based on different categories, where missense mutation, SNP, C > T mutation were the most **(D,E)** Display of TMB in each HCC sample **(F)** The top 10 mutant genes in HCC, including TP53, TTN, CTNNB1, MUC16, ALB, PCLO, MUC, APOB, RYR2, ABCA. **(G)** Landscape of mutation information of each HCC sample in waterfall plot. Each column represents a sample **(H)** Co-expression of mutant genes in HCC. HCC, hepatocellular carcinoma; SNP, single nucleotide polymorphism. *p < 0.001, ˙*p* < 0.05.

We obtained the mutation data of 364 HCC samples processed by “Varsan” from the GDC portal and the “maftools” package was used to visualize the data. Among somatic mutations, missense mutations were the most common type (Figure 1A), single nucleotide polymorphisms and C > T mutations were the most common (Figures 1B,C). The tumor mutation burden of each sample was shown using various colors in box plots (Figures 1D,E). A waterfall plot displays the comprehensive mutation data of each sample . In addition, we selected the 10 most frequently mutated genes in the American HCC’s samples obtained from the TCGA cohort, including *TP53* (30%), *TTN* (24%), *CTNNB1* (25%), *MUC16* (14%), *ALB* (13%), *PCLO* (10%), *MUC* (9%), *APOB* (9%), *RYR2* (9%), and *ABCA* (8%) (Figures 1F,G). Furthermore, we performed co-expression analysis of these mutant genes (Figure 1H).

### Gene Ontology and KEGG Analysis of Gene Expression in the Two TMB Groups

**FIGURE 2:**
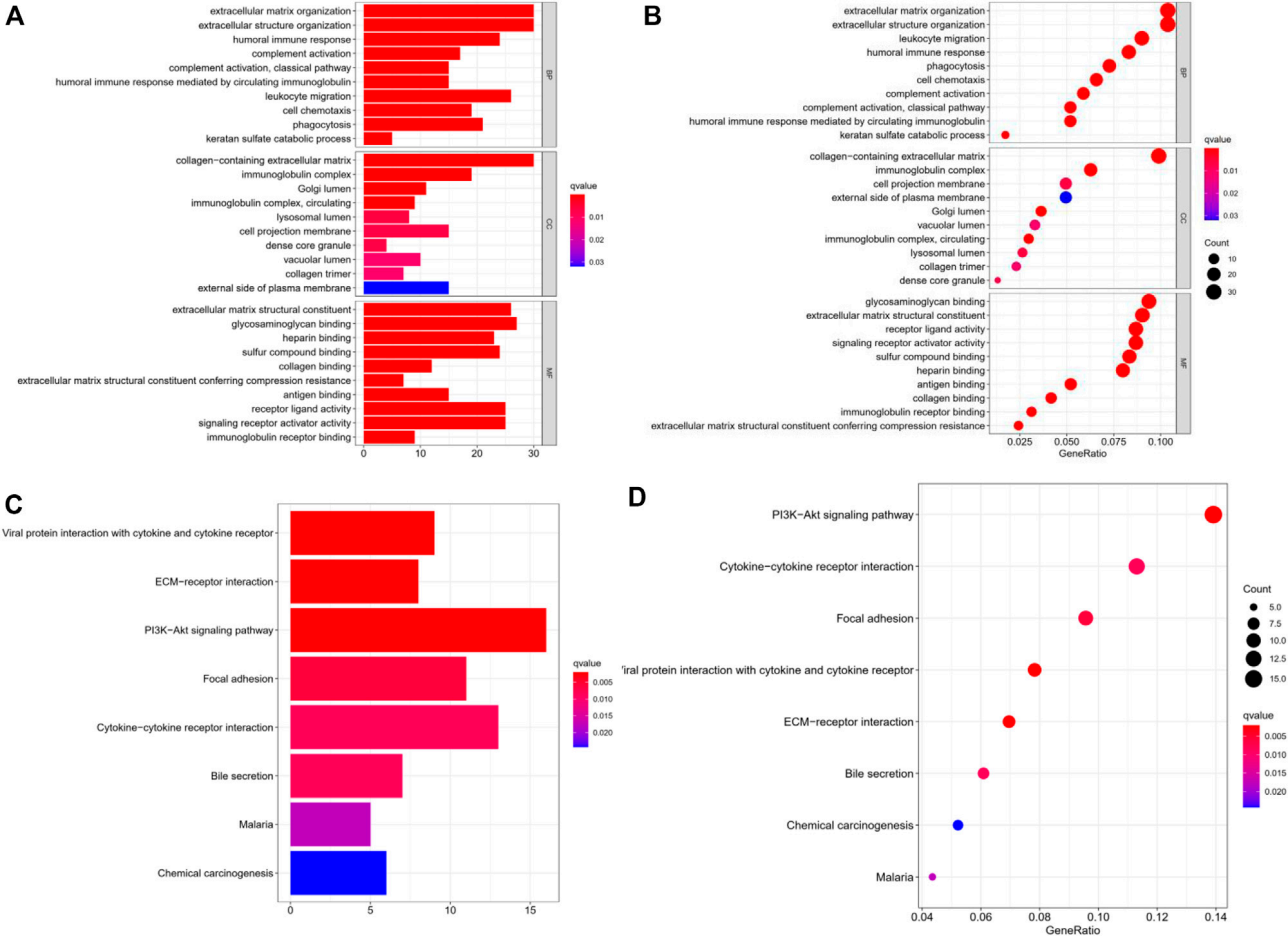
Gene ontology and KEGG analysis of the gene expression of the two TMB groups. **(A,B)** GO enriched analysis of differential genes in BP, CC, MP. **(C,D)** KEGG analysis with these differential genes were enriched in PI3K-AKt, cytokine-cytokine receptor interaction and focal adhesion axis. TMB: tumor mutation burden; BP: biological process; CC, cellular component; MP, molecular function; KEGG, kyoto encyclopedia of genes and genomes.

Differentially expressed genes were obtained through R software (Supplementary Figure S2). The “Limma” package was used to analyze the differentially expressed genes in the two TMB groups, and 375 differentially expressed genes were obtained. Differential genes were identified by log (fold change) > 1 and *p* < 0.05. For the biological processes, we used Gene Ontology to enrich the differential genes, including biological process (BP), cellular component (CC), and molecular function (MF). With regard to BP, these differential genes were mainly enriched in the extracellular matrix organization. With regard to CC and MF, the differential genes were predominantly enriched in the collagen-containing extracellular matrix and matrix structural constituent, respectively (Figures 2A,B). Furthermore, we used KEGG to conduct pathway analysis on these differentially expressed genes, and found that the TMB-related differential genes were mainly enriched in the PI3K-Akt signaling pathway, a pathway that has been clearly reported to be related to cancer (Revathidevi and Munirajan, 2019). In addition, the KEGG analysis revealed that they were also significantly enriched in cytokine-cytokine receptor interaction and focal adhesion pathway (Figures 2C,D; Table 2).

**TABLE 2:** The outcome of KEGG pathway analysis.

Description	GeneRation	p.adj	q	Gene ID
Viral protein interaction with cytokine and cytokine receptor	9/115	0.002	0.002	CXCL6/CCL22/CCL21/CCL2/PF4V1/CCL19/PPBP/IL34/IL6
ECM-receptor interaction	8/115	0.003	0.002	ITGB8/LAMC2/LAMA2/COMP/THBS1/TNXB/ITGB4/THBS2
PI3K-Akt signaling pathway	16/115	0.003	0.002	NTRK2/ITGB8/LAMC2/LAMA2/COMP/FGF1/THBS1/IGF2/TNXB/IL6/IL7R/PDGFD/ITGB4/THBS2/TGFA/VEGFD
Focal adhesion	11/115	0.007	0.006	MYL9/ITGB8/LAMC2/LAMA2/COMP/THBS1/TNXB/PDGFD/ITGB4/THBS2/VEGFD
Cytokine-cytokine receptor interaction	13/115	0.009	0.009	CXCL6/CCL22/CCL21/CCL2/PF4V1/CCL19/PPBP/EDAR/TNFSF15/LTB/IL34/IL6/IL7R
Bile secretion	7/115	0.009	0.009	NCEH1/SCTR/SLC5A1/CFTR/UGT1A3/AQP1/FXYD2
Malaria	5/115	0.020	0.018	CCL2/COMP/THBS1/IL6/THBS2

### Gene Set Enrichment Analysis with High and Low TMB Groups

**FIGURE 3:**
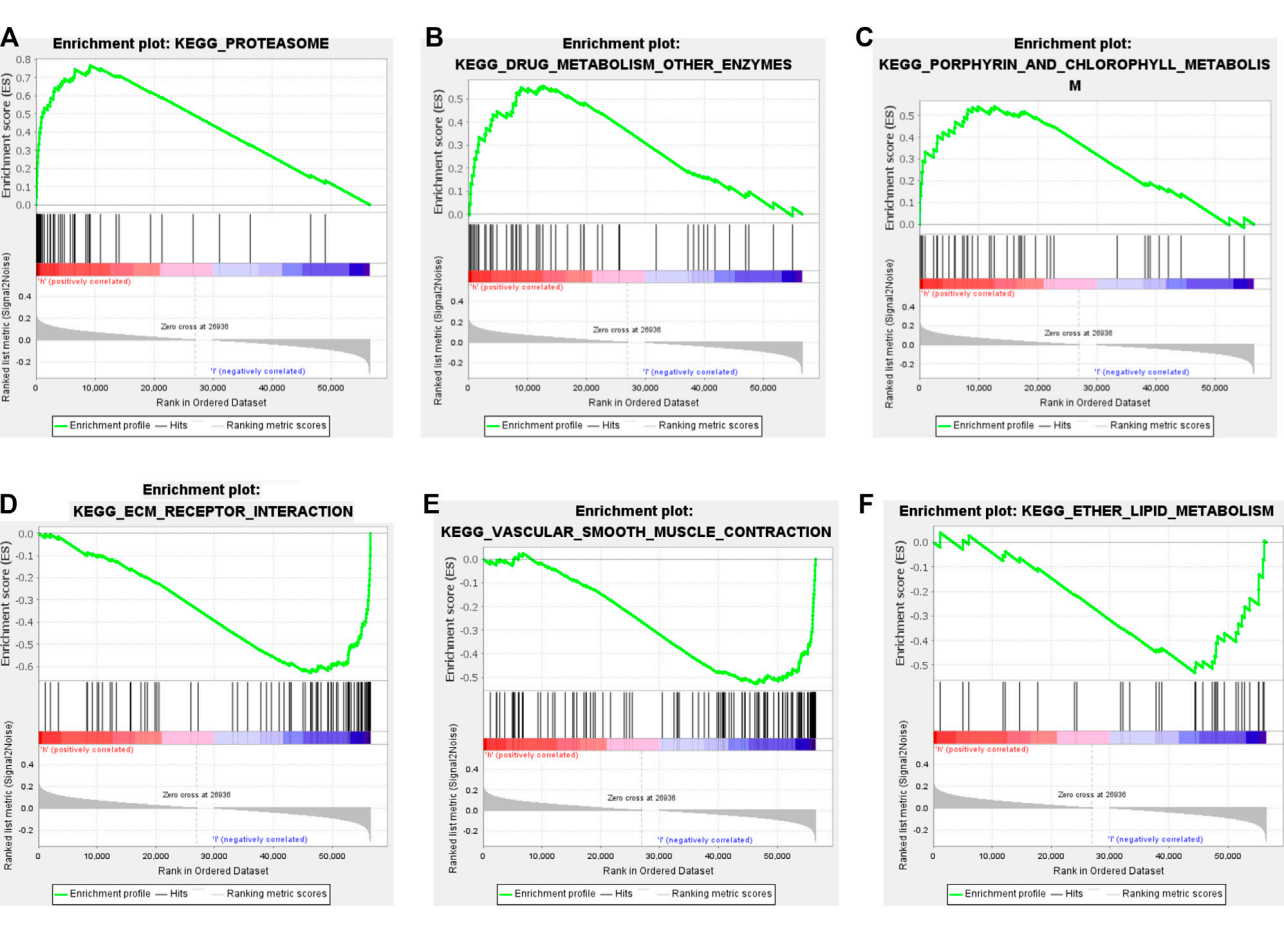
Gene set enrichment analysis GSEA with high and low TMB groups. **(A–C)** The top3 pathway axis enriched in high TMB groups included proteasome, drug metabolism other enzymes, porphyrin and chlorophyll metabolism. **(D–F)** The top3 pathway axis enriched in low TMB groups were ECM receptor interaction, vascular smooth muscle contraction, and ether lipid metabolism.

GSEA was used to analyze the TCGA cohort. We found that in the high TMB group, the differentially expressed genes were mainly enriched in the proteasome, drug metabolism other enzymes, porphyrin, and chlorophyll metabolism (Figures 3A–C). In the low TMB group, the differentially expressed genes chiefly participated in ECM receptor interaction, vascular smooth muscle contraction, and ether lipid metabolism (Figures 3D–F). The top 10 differentially expressed GSEA outcomes in the high and low TMB groups are shown in Supplementary Tables S1, S2.

### Tumor-Infiltrating Immune Cells in Hepatocellular Carcinoma

**FIGURE 4:**
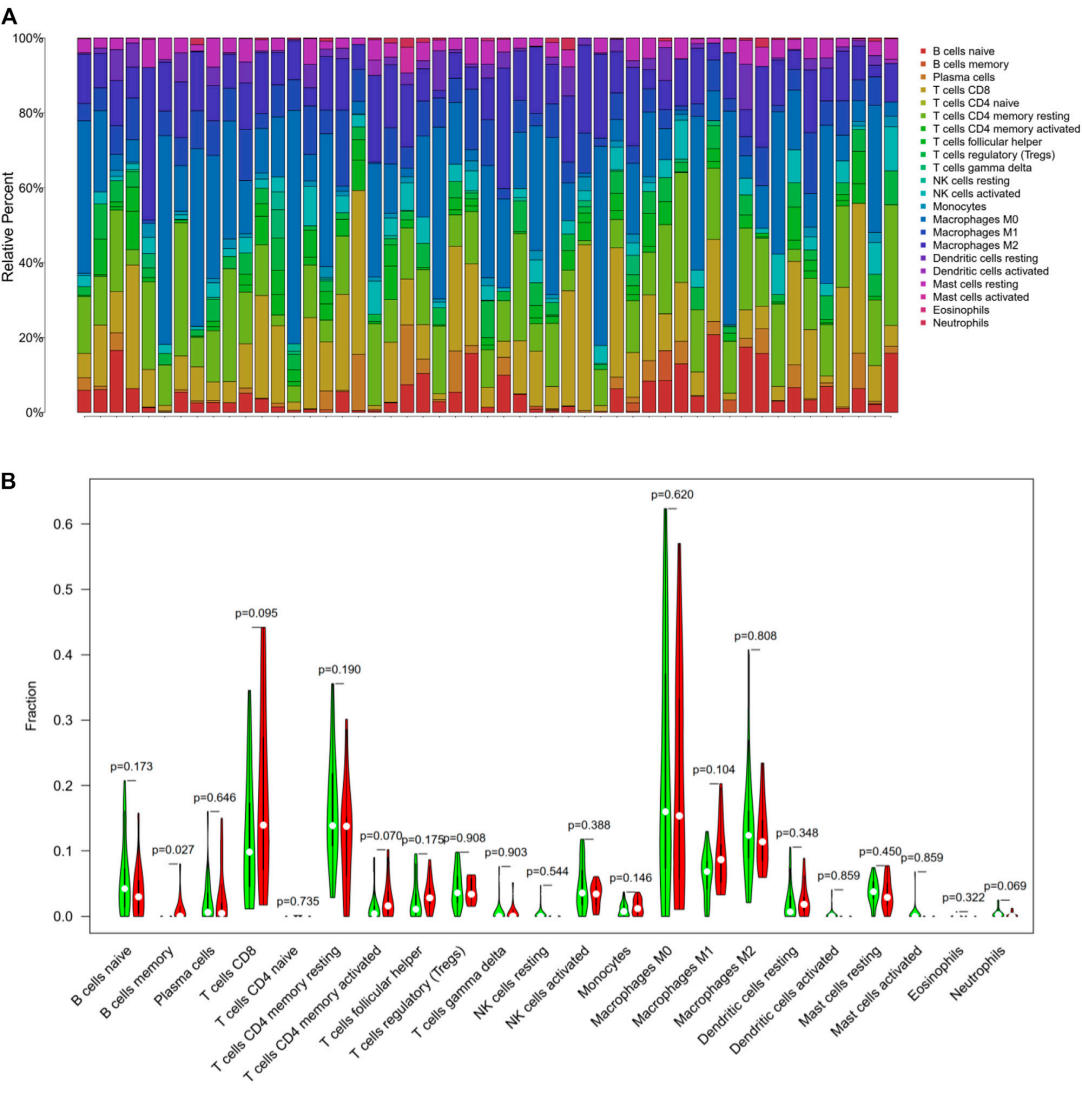
Tumor-infiltrating immune cells in hepatocellular carcinoma. **(A)** The stacked bar graph showed the infiltration of 22 immune cells in each sample. Each color represented a type of immune cell. **(B)** The Wilcoxon rank-sum test displayed that memory B cells (*p* = 0.02), CD8+ T cells (*p* = 0.09), CD4+ memory activated T cells (*p* = 0.07) and neutrophils (*p* = 0.06) had a difference in high and low TMB groups.

Based on the Cibersort algorithm, we analyzed the infiltration of 22 immune cells in the HCC microenvironment. As shown in Figure 4A, each column represents a sample, and different colors represent different immune cells. It was discovered that the 22 kinds of tumor-infiltrating immune cells varied significantly in each sample. Furthermore, we used the R software to process them by dividing them into high and low TMB groups, and compared the immune cell infiltration between the two groups. It was found that the memory B cells (*p* = 0.027) demonstrated a significantly differential expression in the high and low TMB groups. CD8+ T cells (*p* = 0.09), CD4+ memory activated T cells (*p* = 0.07) and neutrophils (*p* = 0.06) also showed differences in expression between the high and low TMB groups (Figure 4B).

### Identification of Significant Immune Genes for HCC Prognostication

**FIGURE 5:**
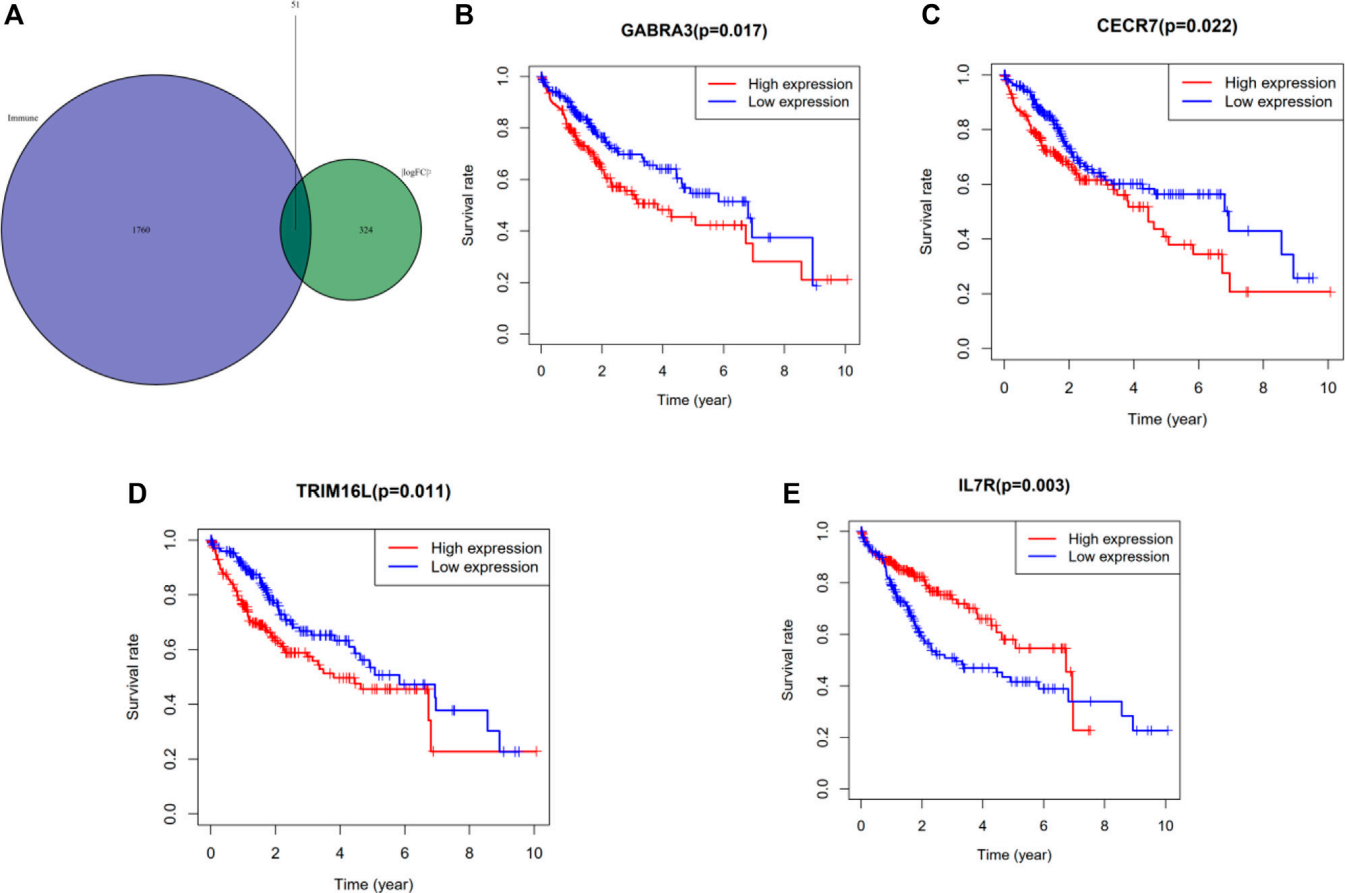
Identification of significant immune genes for HCC prognostication. **(A)** The Venn diagram showed that a total of 51 differential immune genes were associated with tumor mutation burden and immune infiltration. Kaplan–Meier analysis revealed that down-expression of GABRA3, CECR7, TRIM16 and up-expression of IL7R were associated with better survival outcomes and low recurrence. **(B)** GABRA3 (*p* = 0.0017) **(C)** CECR7 (*p* = 0.022) **(D)** TRIM16L (*p* = 0.011) **(E)** IL7R (*p* = 0.003)

Immune-related genes were downloaded from the database (http://www.immport.org/) and intersected with the differential genes in the sample to obtain 51 related differential immune genes (Figure 5A). Subsequently, we performed univariate Cox regression analysis using these differential immune genes, and selected genes with statistical significance for prognosis, including *GABRA3*, *LUCAT1*, *MAGEA12*, *CECR7*, *STEAP4*, *CSAG1*, *TRIM16L*, *LINC00958*, *IL7R*, and *MAGEA3* (Table 3). Multivariate Cox regression analysis was performed as described above and the differential immune genes obtained were *GABRA3*, *CECR7*, *TRIM16L*, and *IL7R*. In addition, we performed survival analysis (Kaplan-Meier method) on these four immune genes and found that the mutations in *GABRA3*, *CECR7*, *TRIM16*, and *IL7R* were related to TMB and promoted anti-tumor immunity. The low expression of *GABRA3*, *CECR7*, and *TRIM16* and the high expression of *IL7R* significantly prolonged the survival time of patients (Figures 5B–E; Table 4). Furthermore, the associations between *GABRA3*, *CECR7*, *TRIM16*, and *IL7R* expression, with immune cell infiltration were shown in Supplementary Figure S3.

**TABLE 3:** Univariate cox analysis of TMB related genes combined with immune infiltrates in HCC.

Gene	HR	HR.95L	HR.95H	Cox *p* value
GABRA3	1.207982	1.09002	1.338709	0.000313
LUCAT1	1.172311	1.084596	1.26712	6.16*E* − 05
MAGEA12	1.032631	1.013041	1.0526	0.001017
CECR7	1.677537	1.391016	2.023075	6.17*E* − 08
STEAP4	0.84268	0.714068	0.994456	0.04279
CSAG1	1.019108	1.006541	1.031832	0.002791
TRIM16L	1.024846	1.010713	1.039177	0.000532
LINC00958	1.144123	1.020469	1.282759	0.021044
IL7R	0.866068	0.768572	0.975932	0.018286
MAGEA3	1.021312	1.008233	1.03456	0.001342

**TABLE 4:** Multivariate cox analysis of TMB related genes combined with immune infiltrates.

Gene	coef	HR	HR.95L	HR.95H	*p* value
GABRA3	0.13787	1.147827	0.997744	1.320485	0.053808
CECR7	0.4248	1.529284	1.245496	1.877735	4.99*E* − 05
TRIM16L	0.021488	1.021721	1.00718	1.036471	0.0033
IL7R	-0.12144	0.885647	0.787232	0.996365	0.043325

### Analysis and Evaluation of the Risk Prediction Model

**FIGURE 6:**
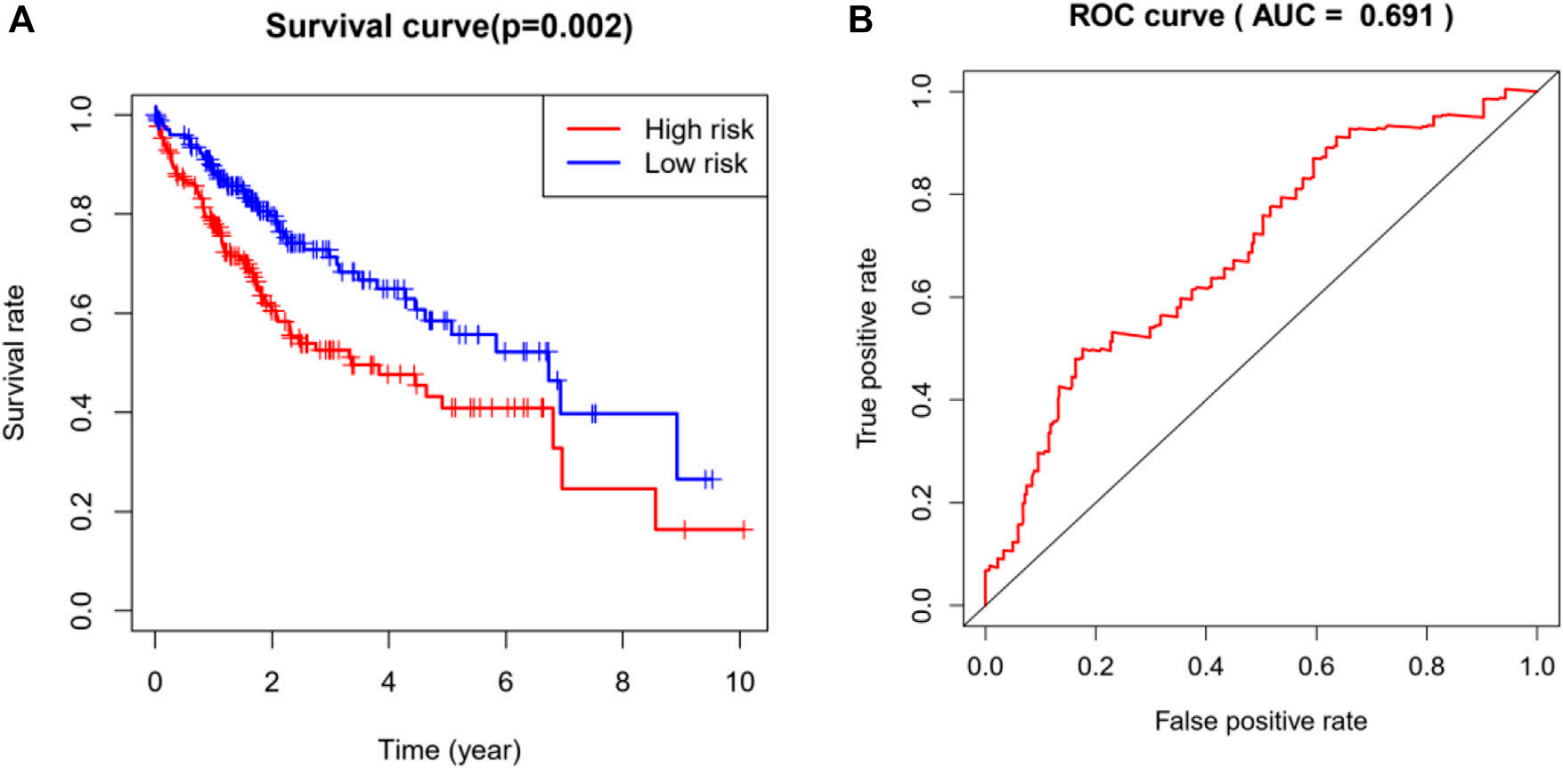
Analysis and evaluation of the risk prediction (diagnosis) models in HCC. **(A)** Kaplan-Meier analysis demonstrated that patients with higher risk showed worse survival rate (*p* = 0.002). **(B)** The AUC of ROC curve (AUC = 0.69) showed the predictive accuracy of TMB risk scores. AUC, area under curve; HCC, hepatocellular carcinoma; ROC, receiver operating characteristic.

Based on the multivariate Cox regression analysis, we constructed a risk prediction model and the coefficients are shown in Table 4. We divided the TCGA cohort into a high-risk group and low-risk group according to the median of the risk scores (median = 0.96), and then performed Kaplan-Meier analysis. It was found that the survival rate of the high-risk group was significantly reduced (*p* = 0.002, Figure 6A). Additionally, by analyzing the ROC curve of the model, we found that the established model had certain clinical significance for the prognosis of HCC (area under the curve [AUC] = 0.691, Figure 6B).

## Discussion

HCC is the fourth leading cause of cancer-related deaths worldwide, with approximately 750,000 new cases each year (Ferlay et al., 2015; Yang et al., 2019). Once HCC is diagnosed, it is often an aggressive or metastatic cancer, and 70% of the patients receive palliative treatment (Llovet et al., 2016; Vogel et al., 2019). The development of HCC is a complex process involving more than 160 genetic changes (Schulze et al., 2015). In recent years, immunotherapy has achieved great success in the treatment of HCC. Immunotherapy is the use of drugs to promote immune cell infiltration in the tumor microenvironment leading to the killing of the tumor cells by T or B lymphocytes. The most common first-line treatment for HCC is sorafenib, which has been proven to prolong the survival of patients with HCC for several months (Kim et al., 2017; Flynn et al., 2019). Immunotherapy mainly includes tumor vaccines, biological therapy, CAR-T cells, and immune checkpoint inhibitors (PD-1, CTLA-7). Liu et al. (2020) reported that the BET protein inhibitor JQ1 can enhance the expression of Rab8A and further upregulate the expression of PD-1, which can enhance the tumor-killing effect of CD8+ cells. In addition to the immune checkpoint inhibitors, tumor vaccines have also been widely used in the treatment of HCC. Lu et al. (2017) indicated that exosomes derived from dendritic cells stimulated the tumor immune response in HCC and killed or inhibited the tumor cells. Tumor immunotherapy has been widely reported to achieve good results in lung cancer, kidney cancer, and melanoma (Larkin et al., 2015).

Although immunotherapy has achieved great success, only a small proportion of patients benefit from it. Previous studies have revealed that patients with a high TMB had accompanying obvious immune cell infiltration and often benefited from immunotherapy. Therefore, there is an urgent need for biomarkers to predict the effect of immunotherapy. Zhu et al. found that the *EP300* mutation was related to the TMB and promoted anti-tumor immunity in bladder cancer (Zhu et al., 2020). Zhang et al. (2020) reported that immune genes such as *CES1* and *CSAG1* could be used as biomarkers for predicting the effects of immunotherapy through a multi-omics analysis. Similarly, Pan et al. (2019) discovered that Laylin is a prognostic marker of survival and is associated with immune infiltration in gastric and colon cancer.

GABRA3 is a type of aminobutyric acid receptor and recognized as a typical oncogene. It is a subunit of ion channel GABAa receptors. Existing studies have shown that GABRA3 is significantly up-regulated in tumor tissues and can regulate tumor proliferation, invasion and metastasis through AKT-mTOR (Long et al., 2017). CECR7 is an immune-related lncRNA. Multi-omics’ study show that it can play an immunomodulatory role through CECR7-hsa-miR-429-CTLA4. Competitive endogenous immune lncRNA CECR7 binds to has-miR-429, regulating the key point of CTLA4 (Yao et al., 2017). TMRI16L and IL7R are also related to tumor immunity, which regulate tumors through the typical JAK/STAT pathway and the endogenous competitive ceRNA network (Fan et al., 2018).

In this study, we downloaded a dataset of 364 American HCC samples from the TCGA. By comparing the immune-infiltrating cells in the high and low TMB groups through the violin chart, we found that memory B cells, CD8+ T cells, and CD4+ memory activated T cells were more abundant in the high TMB group. The main limitation of this study was that we only included samples from the TCGA database and lacked clinical validation experiments. Even if the expressions of GABRA3, CECR7, TRIM16L, and IL7R are obviously changed in HCC in the United States and are associated with TMB and immune infiltration, this does not mean that this risk prediction model can provide a basis for immunotherapy in actual clinical practice. Further clinical trials are needed for verifying this risk prediction model.

In conclusion, our study proved that *GABRA3*, *CECR7*, *TRIM16L*, and *IL7R* were frequently mutated in HCC and related to TMB and immune infiltration. In addition, we built a prognostic model to evaluate the effect of immunotherapy in HCC. These results suggest novel biomarkers for immunotherapy in HCC.

## Data Availability

The datasets presented in this study can be found in online repositories. The names of the repository/repositories and accession number(s) can be found in the article/Supplementary Material.

## Abbreviations

HCC, hepatocellular carcinoma; TMB, tumor mutation burden; TCGA, the cancer genome atlas; GO, gene ontology; GSEA, gene set enrichment analysis; TNM: tumor-node-metastasis; HR: hazard ratio; ECM: extracellular matrix.
