# Supplementary material for: Identification of Tumor Mutation Burden and Immune Infiltrates in Hepatocellular Carcinoma Based on Multi-Omics Analysis
Source: Front Mol Biosci. 2021 Feb 16;7:599142. doi: 10.3389/fmolb.2020.599142 (PMC7928364; doi:10.3389/fmolb.2020.599142)
Supplement: Supplementary file 1 [file table1.docx]

**Table 1. Basic clinical information of all 376 HCC patients from TCGA cohort**

| **Variables** | **TCGA cohorts**  **(n=376)** |
| --- | --- |
| Age | 59.45±13.49 |
| Gender  Female  Male | 122(32.4%)  254(67.6%) |
| Tumor Grade  G1/G2  G3  G4  Unknow | 235(62.5%)  123(32.7%)  13(3.5%)  5(1.3%) |
| Pathologic Stage  I&II  III&IV  Unknow | 261(69.4%)  91(24.2%)  24(6.4%) |
| AJCC-T  T1  T2  T3  T4  Unknow | 185(49.2%)  94(25.0%)  81(21.5%)  13(3.5%)  3(0.8%) |
| AJCC-N  N0  N1-N3  Unknow | 257(68.4%)  4(1.1%)  115(30.6%) |
| AJCC-M  M0  M1  Unknow | 272(72.3%)  4(1.1%)  100(26.6%) |
