# Supplementary material for: Identification of Tumor Mutation Burden and Immune Infiltrates in Hepatocellular Carcinoma Based on Multi-Omics Analysis
Source: Front Mol Biosci. 2021 Feb 16;7:599142. doi: 10.3389/fmolb.2020.599142 (PMC7928364; doi:10.3389/fmolb.2020.599142)
Supplement: Supplementary file 2 [file table2.docx]

**Table 2. The outcome of KEGG pathway analysis**

| **Description** | **GeneRation** | **p.adj** | **q** | **geneID** |
| --- | --- | --- | --- | --- |
| Viral protein interaction with cytokine and cytokine receptor | 9/115 | 0.002 | 0.002 | CXCL6/CCL22/CCL21/CCL2/PF4V1/CCL19/PPBP/IL34/IL6 |
| ECM-receptor interaction | 8/115 | 0.003 | 0.002 | ITGB8/LAMC2/LAMA2/COMP/THBS1/TNXB/ITGB4/THBS2 |
| PI3K-Akt signaling pathway | 16/115 | 0.003 | 0.002 | NTRK2/ITGB8/LAMC2/LAMA2/COMP/FGF1/THBS1/IGF2/TNXB/IL6/IL7R/PDGFD/ITGB4/THBS2/TGFA/VEGFD |
| Focal adhesion | 11/115 | 0.007 | 0.006 | MYL9/ITGB8/LAMC2/LAMA2/COMP/THBS1/TNXB/PDGFD/ITGB4/THBS2/VEGFD |
| Cytokine-cytokine receptor interaction | 13/115 | 0.009 | 0.009 | CXCL6/CCL22/CCL21/CCL2/PF4V1/CCL19/PPBP/EDAR/TNFSF15/LTB/IL34/IL6/IL7R |
| Bile secretion | 7/115 | 0.009 | 0.009 | NCEH1/SCTR/SLC5A1/CFTR/UGT1A3/AQP1/FXYD2 |
| Malaria | 5/115 | 0.020 | 0.018 | CCL2/COMP/THBS1/IL6/THBS2 |
