# Supplementary material for: Identification of Tumor Mutation Burden and Immune Infiltrates in Hepatocellular Carcinoma Based on Multi-Omics Analysis
Source: Front Mol Biosci. 2021 Feb 16;7:599142. doi: 10.3389/fmolb.2020.599142 (PMC7928364; doi:10.3389/fmolb.2020.599142)
Supplement: Supplementary file 3 [file table3.docx]

**Table 3. Univariate Cox analysis of TMB related genes combined with immune infiltrates in HCC**

| **Gene** | **HR** | **HR.95L** | **HR.95H** | **Cox P value** |
| --- | --- | --- | --- | --- |
| GABRA3 | 1.207982 | 1.09002 | 1.338709 | 0.000313 |
| LUCAT1 | 1.172311 | 1.084596 | 1.26712 | 6.16E-05 |
| MAGEA12 | 1.032631 | 1.013041 | 1.0526 | 0.001017 |
| CECR7 | 1.677537 | 1.391016 | 2.023075 | 6.17E-08 |
| STEAP4 | 0.84268 | 0.714068 | 0.994456 | 0.04279 |
| CSAG1 | 1.019108 | 1.006541 | 1.031832 | 0.002791 |
| TRIM16L | 1.024846 | 1.010713 | 1.039177 | 0.000532 |
| LINC00958 | 1.144123 | 1.020469 | 1.282759 | 0.021044 |
| IL7R | 0.866068 | 0.768572 | 0.975932 | 0.018286 |
| MAGEA3 | 1.021312 | 1.008233 | 1.03456 | 0.001342 |
