# Supplementary material for: Identification of Tumor Mutation Burden and Immune Infiltrates in Hepatocellular Carcinoma Based on Multi-Omics Analysis
Source: Front Mol Biosci. 2021 Feb 16;7:599142. doi: 10.3389/fmolb.2020.599142 (PMC7928364; doi:10.3389/fmolb.2020.599142)
Supplement: Supplementary file 4 [file table4.docx]

**Table 4. Multivariate cox analysis of TMB related genes combined with immune infiltrates**

| **Gene** | **coef** | **HR** | **HR.95L** | **HR.95H** | **pvalue** |
| --- | --- | --- | --- | --- | --- |
| GABRA3 | 0.13787 | 1.147827 | 0.997744 | 1.320485 | 0.053808 |
| CECR7 | 0.4248 | 1.529284 | 1.245496 | 1.877735 | 4.99E-05 |
| TRIM16L | 0.021488 | 1.021721 | 1.00718 | 1.036471 | 0.0033 |
| IL7R | -0.12144 | 0.885647 | 0.787232 | 0.996365 | 0.043325 |
