# Supplementary material for: Identification of Tumor Mutation Burden and Immune Infiltrates in Hepatocellular Carcinoma Based on Multi-Omics Analysis
Source: Front Mol Biosci. 2021 Feb 16;7:599142. doi: 10.3389/fmolb.2020.599142 (PMC7928364; doi:10.3389/fmolb.2020.599142)
Supplement: Supplementary file 5 [file table5.docx]

**Table S1: The top10 GSEA outcomes in high TMB**

| **Description** | **size** | **ES** | **NES** | **p** | **q** | **Rank at max** |
| --- | --- | --- | --- | --- | --- | --- |
| Proteasome | 46 | 0.77 | 1.98 | 0.004 | 0.035 | 9185 |
| Drug metabolism other enzymes | 51 | 0.56 | 1.75 | 0.008 | 0.28 | 12650 |
| Porphyrin and chlorophyll metabolism | 41 | 0.54 | 1.65 | 0.022 | 0.448 | 12650 |
| Amino sugar and nucleotide sugar metabolism | 44 | 0.51 | 1.58 | 0.034 | 0.598 | 6515 |
| RNA polymerase | 29 | 0.6 | 1.57 | 0.051 | 0.504 | 14603 |
| Peroxisome | 78 | 0.52 | 1.53 | 0.07 | 0.564 | 8401 |
| Aminoacyl tRNA biosynthesis | 41 | 0.57 | 1.51 | 0.084 | 0.564 | 15112 |
| Glutathione metabolism | 49 | 0.45 | 1.49 | 0.048 | 0.561 | 10063 |
| Metabolism of xenobiotics by cytochrome P450 | 69 | 0.5 | 1.49 | 0.068 | 0.505 | 13033 |
| N-glycan biosynthesis | 46 | 0.5 | 1.48 | 0.056 | 0.479 | 10599 |
