# Supplementary material for: Identification of Tumor Mutation Burden and Immune Infiltrates in Hepatocellular Carcinoma Based on Multi-Omics Analysis
Source: Front Mol Biosci. 2021 Feb 16;7:599142. doi: 10.3389/fmolb.2020.599142 (PMC7928364; doi:10.3389/fmolb.2020.599142)
Supplement: Supplementary file 6 [file table6.docx]

**Table S2. The top10 GSEA outcomes in low TMB**

| **Description** | **size** | **ES** | **NES** | **p** | **q** | **Rank at max** |
| --- | --- | --- | --- | --- | --- | --- |
| ECM receptor interaction | 84 | -0.63 | -1.89 | 0.000 | 0.139 | 10194 |
| Vascular smooth muscle contraction | 115 | -0.53 | -1.77 | 0.004 | 0.296 | 10325 |
| Ether lipid metabolism | 33 | -0.53 | -1.71 | 0.002 | 0.339 | 12106 |
| Taste transduction | 51 | -0.54 | -1.7 | 0.004 | 0.279 | 11197 |
| Cytokine-cytokine receptor interaction | 264 | -0.51 | -1.69 | 0.008 | 0.246 | 10851 |
| GNRH signaling pathway | 101 | -0.51 | -1.68 | 0.010 | 0.242 | 12095 |
| Focal adhesion | 199 | -0.53 | -1.66 | 0.014 | 0.229 | 9255 |
| Dilated cardiomyopathy | 90 | -0.53 | -1.66 | 0.010 | 0.214 | 12404 |
| JAK-STAT signaling pathway | 155 | -0.5 | -1.65 | 0.014 | 0.207 | 15883 |
| DORSO ventral axis formation | 24 | -0.6 | -1.63 | 0.011 | 0.214 | 2959 |
